# Liquid-crystalline half-Skyrmion lattice spotted by Kossel diagrams

**DOI:** 10.1038/s41598-018-35514-0

**Published:** 2018-11-22

**Authors:** Jun-ichi Fukuda, Andriy Nych, Uliana Ognysta, Slobodan Žumer, Igor Muševič

**Affiliations:** 1 Department of Physics, Kyushu University, 744, Motooka, Nishi-ku, Fukuoka, 819-0395 Japan; 2 National Institute of Advanced Industrial Science and Technology (AIST), 1-1-1 Umezono, Tsukuba, 305-8568 Japan; 3 Faculty of Mathematics and Physics, University of Ljubljana, Jadranska 19, SI-1000 Ljubljana, Slovenia; 4 Department of Molecular Photoelectronics, Institute of Physics, prospect Nauky, 46, Kyiv, 03680 Ukraine; 5 Condensed Matter Department, Jožef Stefan Institute, Jamova 39, SI-1000 Ljubljana, Slovenia

## Abstract

Skyrmions are swirl-like topological entities that have been shown to emerge in various condensed matter systems. Their identification has been carried out in different ways including scattering techniques and real-space observations. Here we show that Kossel diagrams can identify the formation of a hexagonal lattice of half-Skyrmions in a thin film of a chiral liquid crystal, in which case Kossel lines appear as hexagonally arranged circular arcs. Our experimental observations on a hexagonal lattice of half-Skyrmions and other defect structures resembling that of a bulk cholesteric blue phase are perfectly accounted for by numerical calculations and a theoretical argument attributing strong reflections yielding Kossel lines to guided mode resonances in the thin liquid crystal film. Our study demonstrates that a liquid crystal is a model system allowing the investigation of topological entities by various optical means, and also that Kossel techniques are applicable to the investigation of thin systems with non-trivial photonic band structures including topologically protected optical surface states.

## Introduction

Skyrmions are not real particles with distinct physical properties, but coreless solitonic field excitations that behave like a particle. Skyrmions were originally proposed to explain the emergence of particle-like entities in a continuous field theory^1^. Now Skyrmions have been shown to exist in a wide variety of condensed matter systems characterised by vectorial order parameter(s), including two-dimensional electron gases^2–4^, spinor Bose-Einstein condensates^5,6^, superfluid He^3^-*A* phase^7–9^, and chiral liquid crystals^10–16^. Not only have Skyrmions attracted interest from an academic point of view as a realisation of non-trivial topological entities that can be classified by second homotopy groups, those appearing in chiral ferromagnets^17–26^ have been extensively studied because of the possibility of practical applications in high-density information storage and manipulation of electrons^27,28^.

Skyrmions in liquid crystals, the subject of our study, and magnetic ones have many commonalities. Both are described phenomenologically by a vector order parameter (in magnetic systems the vector magnetisation ***m***, and in liquid crystals the director ***n***, a unit vector without head-tail distinction, that allows the existence of additional topologically distinct structures). The chirality in both systems manifests itself in the Lifshitz invariant of the form ***n***⋅∇ × ***n*** (and the same with ***m*** for magnetic systems) in the free energy that stabilises Skyrmions^17,18^. Our study concerns a hexagonal lattice of half-Skyrmions in which the order parameter ***n***(*x*, *y*) in the two-dimensional plane (*x*, *y*) rotates by *π*/2 from the Skyrmion centre to its perimeter, and the the Skyrmion number $N=\mathrm{(1/4}\pi )\int dxdy{\boldsymbol{n}}\cdot (\partial {\boldsymbol{n}}/\partial x\times \partial {\boldsymbol{n}}/\partial y)$ is ±1/2 (the sign of a Skyrmion number is meaningless in the case of a liquid crystal because of the head-tail symmetry). We have shown^12,16^ that a chiral liquid crystal whose helical pitch is a few hundred nanometers can exhibit a hexagonal lattice of half-Skyrmions, each surrounded by six topological defects of winding number −1/2. When only the orientational order at the perimeter is concerned, a half-Skyrmion can be regarded as an entity with winding number +1 that can be compensated by −1/2 defects twice as many as half-Skyrmions. Because half-integer defects are allowed only for vectorial order parameter without head-tail distinction, our half-Skyrmion lattice does not have an exact magnetic counterpart. Instead, magnetic half-Skyrmions form a square lattice with −1 defects in between^19,24^, or a hexagonal lattice where each half-Skyrmion is surrounded by six topological charges with winding number −1 and three smaller triangular regions with the magnetisation opposite to that of the half-Skyrmion centre^25^. Half-Skyrmions in a ferromagnet have been also shown to arise in a precursor state^22^.

Identification of the formation of Skyrmions is highly important in the experimental studies of Skyrmions, and it has been carried out in many different ways depending on the system studied. Direct real-space observation of ferromagnetic Skyrmions by Lorentz transmission electron microscopy^23^ ignited the field of “Skyrmionics,” and real-space identification of half-Skyrmions in a chiral liquid crystal was realised by conventional, although with high numerical apertures, optical microscopy^16^. However, the first identification of ferromagnetic Skyrmions was by neutron scattering^20^ (together with the measurement of the Hall effect for which Skyrmions are responsible)^21^ to demonstrate that they form a hexagonal lattice. The identification of Skyrmions as many different ways as possible can corroborate their formation in a more convincing manner, and enables the investigation of various aspects of their structural and dynamical properties.

Here we demonstrate that the formation of a hexagonal lattice of half-Skyrmions in a thin film of a chiral liquid crystal, previously identified by optical microscopy, is confirmed also by Kossel diagrams using visible light. Kossel diagrams or Kossel lines visualise the directions of strong reflections or diffractions of incident monochromatic wave (visible light, X-ray, electrons etc.)^29^. Kossel lines, also referred to as Kikuchi lines for electron microscopy^30^, provide information on the symmetry of bulk crystals, and have been applied to the identification of not only solid crystals but also soft matter systems including crystallised colloidal suspensions^31–33^. Note that Kossel diagrams played a decisive role in the investigation of the symmetry and the structural changes of then-mysterious cholesteric blue phases of a chiral liquid crystal^34,35^. Kossel diagrams are commonly used for bulk crystals and then Bragg reflections are responsible for Kossel lines. We show that Kossel lines are observed also for our thin system where Kossel lines cannot be simply attributed to Bragg reflections, and that the Kossel lines should be attributed to the dispersion modes that are present only in finite systems with surfaces.

## Results

### Experimental Kossel diagrams

**Figure 1:**
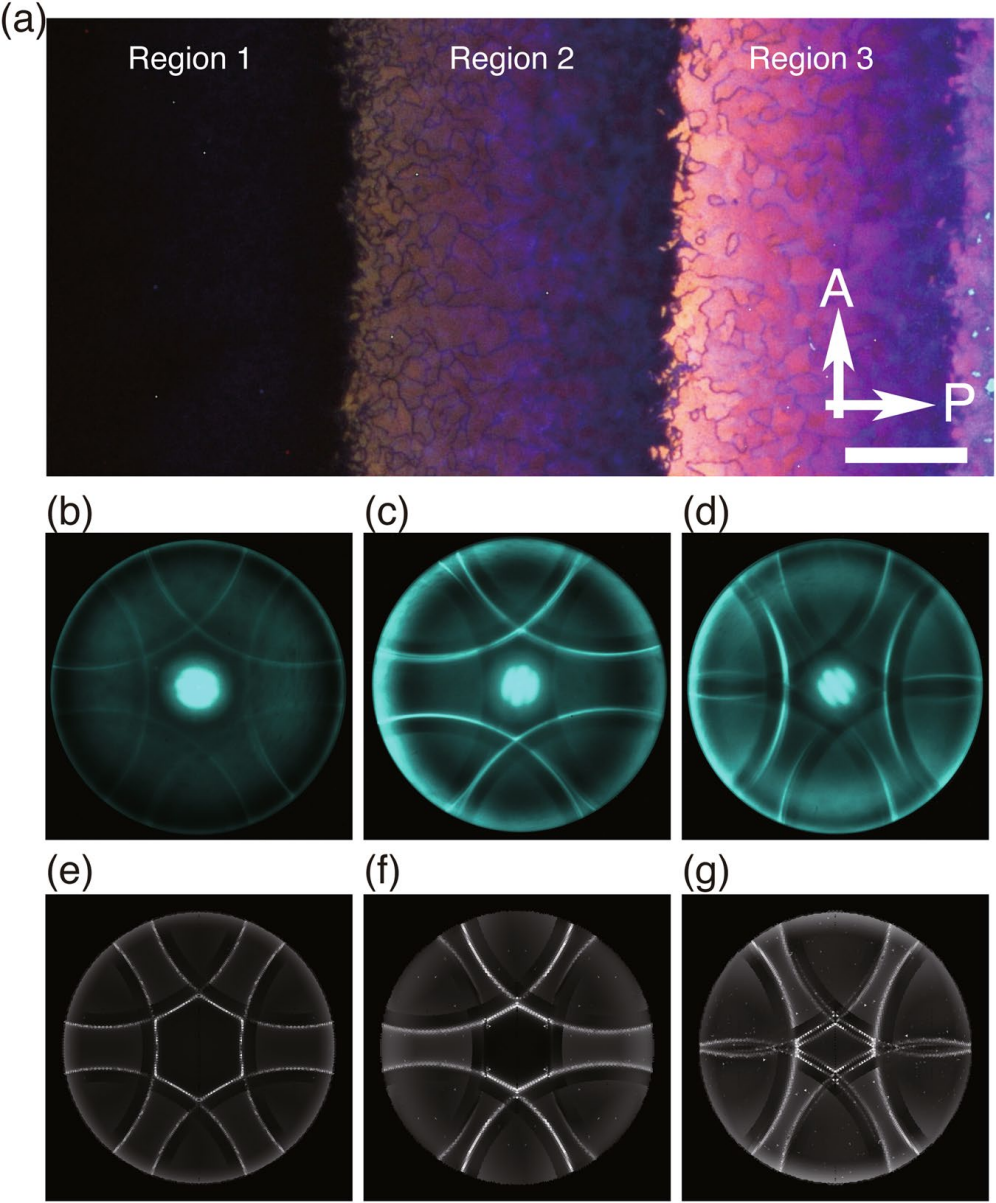
Kossel diagrams. (**a**) Colour image of the texture of a wedge cell under a polarising microscope. The directions of the polariser (**P**) and the analyser (**A**) are depicted by arrows. Cell thickness is smaller to the left. Scale bar: 50 μm. Reproduced from ref.^16^. (**b–d**) Experimentally obtained Kossel diagrams for “Region 1”, “Region 2” and “Region 3”, respectively, at 514.5 nm. Central bright spots, not present in the following numerically calculated Kossel diagrams, are artifacts that arise due to parasitic reflections from glass–liquid crystal and glass–immersion oil interfaces along the sample normal. (**e–g**) Numerically calculated Kossel diagrams for the structures shown in Fig. 2(a,b), Fig. 2(c,d) and Fig. 2(e,f), respectively. The ratio *λ*/*p* is taken equal to that of the experiments.

As in our previous work^16^, we studied a wedge cell of a chiral liquid crystal exhibiting a stable cubic blue phase known as BP I whose lattice constant is $a\simeq 360$ nm (Fig. 1(a). See Methods for more details). We demonstrated the formation of a hexagonal lattice of half-Skyrmions in a thin region (thickness $\lesssim 250$–260 nm, referred to as “Region 1” in ref.^16^), and a structure resembling a thin slice of the cubic lattice of BP I (“Region 2” neighbouring Region 1, with thickness between $\simeq 250$–260 nm and $\simeq 430$ nm). In the present study, in addition to Regions 1 and 2, we also investigate “Region 3” with thickness between $\simeq 430$ nm and $\simeq 560$–570 nm adjacent to Region 2.

For a single domain from the Regions, we studied the Kossel diagrams visualising the directions of strongly reflected light when the sample is illuminated by monochromatic light from a range of incident directions^35^. Experimentally obtained Kossel diagrams for Regions 1, 2 and 3 are shown in Fig. 1(b–d). The wavelength of the incident light was *λ* = 514.5 nm (See Methods). One can clearly see the six-fold symmetry in the Kossel diagram in Fig. 1(b) for Region 1. The six-fold symmetry of the Kossel diagram is consistent with the hexagonal symmetry of the half-Skyrmion lattice we identified earlier^16^. We also find a pronounced difference between the Kossel diagrams for Regions 2 and 3 (Fig. 1(c,d)) and those for the bulk BP I (See Supplementary Fig. 1(d) of ref.^16^); Kossel lines of the former are circular arcs as we will see below, while those of the latter contain ellipses.

### Numerical calculations

**Figure 2:**
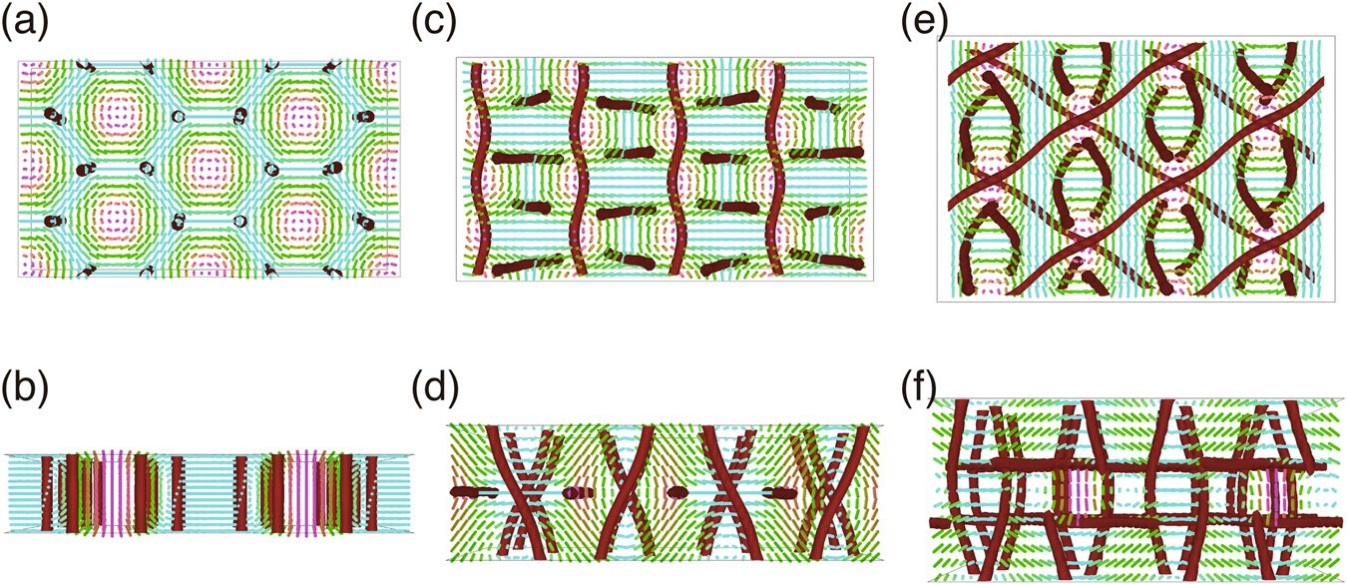
Numerically calculated real-space profiles of liquid crystals. **(a**,**b)** Profile of a hexagonal half-Skyrmion lattice in a cell of thickness *L* = 0.557*p*, where *p* is the natural pitch of the helical orientational order. (**c**,**d)** Structure similar to a sliced BP I containing one in-plane array of parallel disclination lines in a cell of thickness *L* = 0.955*p*. (**e**,**f)** Structure similar to a sliced BP I containing two in-plane arrays of parallel disclination lines in a cell of thickness *L* = 1.273*p*. Thick red lines are disclination lines. In the top views in (**a**,**c**,**e**) the orientational order at the midplane are represented by short rods whose colouring is such that it is blue (magenta) when the orientational order is parallel (perpendicular) to the cell. (**b**,**d**,**f)** Present the perspective view with the orientation profile at some vertical plane.

In our previous study^16^, we carried out numerical calculations of the orientational order of the liquid crystal to show that a hexagonal lattice of half-Skyrmions (Fig. 2(a,b)) and a structure resembling a bulk BP I sliced by two [110] planes (Fig. 2(c,d)) are formed in Regions 1 and 2, respectively. These structures accommodate topological line defects of orientational order, or disclination lines, as bulk cholesteric blue phases do. Further calculations (See Supplementary Methods for details) revealed that a thicker slab of cholesteric blue phase I sliced by the same [110] planes is stable at larger thicknesses (Fig. 2(e,f)). A calculated phase diagram is presented in Supplementary Fig. 1(a), and see Supplementary Fig. 1(b) for another similar stable structure resembling bulk BP I sliced by the same planes. Although bulk BP I liquid crystals sandwiched by two untreated glass plates are known to exhibit polydomain textures, [110] orientation of the cubic lattice is one of the commonly observed orientations^36–38^.

The typical lattice spacing of half-Skyrmions is $\simeq 275$ nm (ref.^16^), of the order of or smaller than the wavelength in the liquid crystal medium (its average refractive index is $\simeq 1.6$). Therefore, to discuss the optical properties of our half-Skyrmion lattice and other structures, geometrical optics is totally useless, and we have to solve the full Maxwell equations for light waves. See ref.^39^ for technical details of the calculation, and the ratio of *λ* to the natural pitch of the helical orientational order *p* (approximately equal to *a*, the lattice constant of BP I) was chosen to be equal to that of the experiments (=514.5 nm/360 nm). In Fig. 1(e–g), we show the Kossel diagrams numerically calculated as such for the orientation profiles in Fig. 2. Most of the qualitative features found in the experimental Kossel diagrams are successfully reproduced in the numerically calculated ones, including the hexagonal nature of the Kossel diagrams for a thin system corresponding to Region 1 (Fig. 1(b,e)), the strong intensity difference of the Kossel lines for Regions 2 and 3 (Fig. 1(c,f) and Fig. 1(d,g) respectively), higher intensity of the Kossel lines for Regions 2 and 3 than for Region 1, the presence of faint filled areas inside the sharp Kossel lines, and the circular shape of the Kossel lines. The close similarity between the experimental and the numerically calculated Kossel diagrams, along with our earlier real-space observations^16^, supports the correspondence between the structures found in the experiments and those obtained in the numerical calculations (Fig. 2).

### Theoretical interpretation of the Kossel lines

Kossel technique is commonly used for the identification of the structure of bulk crystals, in which case Bragg reflections are responsible for Kossel lines. In our case of a thin system without a well-defined 3D lattice, however, Kossel lines cannot be attributable to Bragg reflections. We also note that Kossel lines of circular arc cannot be accounted for by Bragg reflections because a naive construction of a Kossel diagram of a 2D hexagonal lattice structure based on the projection of “Kossel cones”^32^ would result in six straight lines forming a hexagon, which obviously contradicts our circular Kossel diagram.

Here we show that Kossel lines for a thin system arise from guided-mode resonances and interactions between different modes through the periodic medium, known to be responsible for Wood anomalies in diffraction gratings^40,41^. We follow the discussion in ref.^41^ on the guided-mode resonances in a planar diffraction grating, and consider a slab of thickness *L* with weak spatial modulation of the dielectric tensor ${\epsilon }_{\alpha \beta }$ along the in-plane direction. The dielectric constant of the surrounding medium is set to ${\epsilon }_{1}$, and the average of ${\epsilon }_{\alpha \beta }$ inside the slab is assumed to be ${\epsilon }_{2}$*δ*_*αβ*_, where ${\epsilon }_{2}$ > ${\epsilon }_{1}$.

We let *k*_*p*_ denote the wavenumber of the guided wave along the in-plane direction of the slab, and ${k}_{1}=\sqrt{{\epsilon }_{1}}\omega /c$ the wavenumber of light in the surrounding medium (*ω* is the angular frequency and *c* is the speed of light in vacuum). For the sake of brevity in the following, we define $\kappa \equiv \sqrt{({\epsilon }_{2}/{\epsilon }_{1}){k}_{1}^{2}-{k}_{p}^{2}}$ and $\gamma =\sqrt{{k}_{p}^{2}-{k}_{1}^{2}}$. In the limit of vanishing spatial modulation of ${\epsilon }_{\alpha \beta }$ inside the slab, *k*_*p*_ must satisfy^42^

1 $$1\le \frac{{k}_{p}}{{k}_{1}} < \sqrt{\frac{{\epsilon }_{2}}{{\epsilon }_{1}}}$$

(that is, *κ* > 0 and *γ* ≥ 0), and

2 $$\begin{array}{llll}\tan \,\kappa L & = & \frac{2\kappa \gamma }{{\kappa }^{2}-{\gamma }^{2}} & [{\rm{for}}\,{\rm{transverse}}\,{\rm{electric}}\,({\rm{TE}})\,{\rm{mode}}]\\ \tan \,\kappa L & = & \frac{2{\epsilon }_{1}{\epsilon }_{2}\kappa \gamma }{{\epsilon }_{1}^{2}{\kappa }^{2}-{\epsilon }_{2}^{2}{\gamma }^{2}}\,\, & [{\rm{for}}\,{\rm{transverse}}\,{\rm{magnetic}}\,({\rm{TM}})\,{\rm{mode}}]\end{array}$$

for a guided wave mode to exist. In our theoretical case (See Supplementary Methods), ${\epsilon }_{1}$ = 2.25, ${\epsilon }_{2}$ = 2.571, and ${k}_{1}=2\pi \sqrt{{\epsilon }_{1}}/\lambda =\mathrm{6.597/}p$. We denote *k*_*p*_ for TE mode and TM mode by *k*_*p*_^TE^ and *k*_*p*_^TM^, respectively, and Table 1 summarises *k*_*p*_^TE^ and *k*_*p*_^TM^ (in units of *p*^−1^) calculated from eqs. (1) and (2) for systems with different *L* shown in Fig. 2. Note that there are two solutions for *k*_*p*_^TM^ when *L*/*p* = 1.273.

**Table 1:** Wavenumber of the guided modes for systems with different thickness.

	*L*/*p*	k_*p*_^TE^ *p*	k_*p*_^TM^ *p*
Fig. 2(a,b)	0.557	6.737	6.719
Fig. 2(c,d)	0.955	6.842	6.826
Fig. 2(e,f)	1.273	6.897	6.597 and 6.884

**Figure 3:**
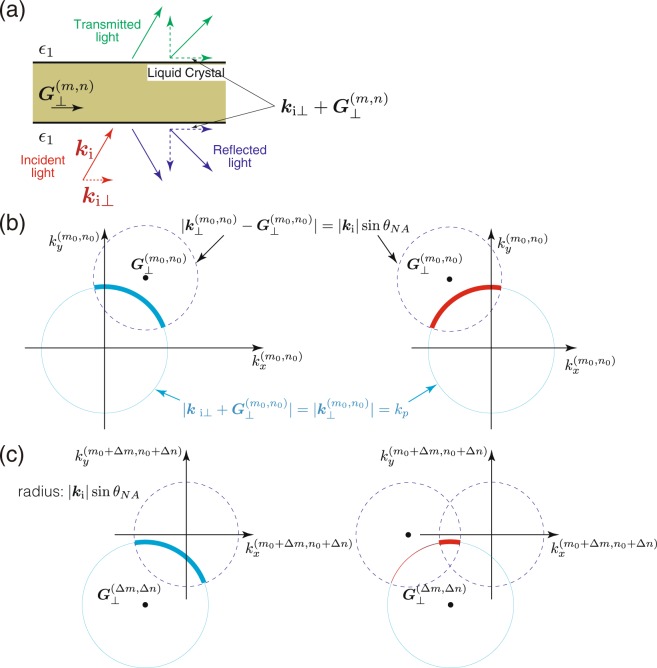
Illustration of the theoretical argument on Kossel diagrams. **(a)** Schematic illustration of the geometry of the system and relevant wavevectors. (**b**,**c)** Illustration explaining how a Kossel line becomes a circular arc (See text). In the left, ${{\bf{G}}}_{\perp }^{({m}_{0},{n}_{0})}+{{\boldsymbol{G}}}_{\perp }^{({\rm{\Delta }}m,{\rm{\Delta }}n)}=0$ and in the right, ${{\bf{G}}}_{\perp }^{({m}_{0},{n}_{0})}+{{\bf{G}}}_{\perp }^{({\rm{\Delta }}m,{\rm{\Delta }}n)}\ne 0$. The overlap of two dotted circles in the right panel of (c) corresponds to a faint filled area.

Let ***k***_i_ and ***k***_i⊥_ denote the wavevector of the incident light and its in-plane component, respectively (Fig. 3(a)), and |***k***_i⊥_| < |***k***_i_| sin*θ*_*NA*_, where *θ*_*NA*_ is determined by the numerical aperture of the objective lens *NA* so that $\sqrt{{\epsilon }_{1}}\,\sin \,{\theta }_{NA}=NA$. When the in-plane structure of the liquid crystal is characterised by the 2D reciprocal lattice vector ${{\boldsymbol{G}}}_{\perp }^{(m,n)}$ labelled by two integers (*m*, *n*), the in-plane component of the wavevector of the reflected light is, because of the Bloch theorem, ${{\boldsymbol{k}}}_{i\perp }+{{\boldsymbol{G}}}_{\perp }^{(m,n)}\equiv {{\boldsymbol{k}}}_{\perp }^{(m,n)}$ (See Fig. 3(a)). Kossel lines represent ${{\boldsymbol{k}}}_{\perp }^{(m,n)}$ of the reflected light with strong reflectivity^32^, and $|{{\boldsymbol{k}}}_{\perp }^{(m,n)}|$ must be smaller than |***k***_i_| sin*θ*_*NA*_, because in the experimental setup the same objective lens collects the reflected light.

Note that ${k}_{p}=|{{\boldsymbol{k}}}_{i\perp }|(\,=\,|{{\boldsymbol{k}}}_{\perp }^{\mathrm{(0,0)}}|)$ cannot satisfy eq. (1) because |***k***_i⊥_| < |***k***_i_| sin*θ*_*NA*_ < |***k***_i_| = *k*_1_, which implies the inability for the incident wave to excite the guided mode in an unmodulated slab^42^. However, the periodic modulation of ${\epsilon }_{\alpha \beta }$ in the slab gives rise to the interaction between modes with different (*m*, *n*), and there exists (*m*_0_, *n*_0_) that satisfies $|{{\boldsymbol{k}}}_{{\rm{i}}\perp }+{{\boldsymbol{G}}}_{\perp }^{({m}_{0},{n}_{0})}|(\,=\,|{{\boldsymbol{k}}}_{\perp }^{({m}_{0},{n}_{0})}|)={k}_{p}$ (here *k*_*p*_ is the solution to eq. (2)) for a given continuous set of ***k***_i⊥_. This set of $|{{\boldsymbol{k}}}_{\perp }^{({m}_{0},{n}_{0})}|$, in-plane wavevector of the excited guided mode, is a part of a circle (or an arc) of radius *k*_*p*_ inside another circle $|{{\boldsymbol{k}}}_{\perp }^{({m}_{0},{n}_{0})}-{{\boldsymbol{G}}}_{\perp }^{({m}_{0},{n}_{0})}|(\,=\,|{{\boldsymbol{k}}}_{i\perp }|) < |{{\boldsymbol{k}}}_{i}|\,\sin \,{\theta }_{NA}$ (Fig. 3(b)). This excited mode with the in-plane wavenumber being ${{\boldsymbol{k}}}_{\perp }^{({m}_{0},{n}_{0})}$ couples to different modes with ${{\boldsymbol{k}}}_{\perp }^{({m}_{0}+{\rm{\Delta }}m,{n}_{0}+{\rm{\Delta }}n)}={{\boldsymbol{k}}}_{\perp }^{({m}_{0},{n}_{0})}+{{\boldsymbol{G}}}_{\perp }^{({\rm{\Delta }}m,{\rm{\Delta }}n)}={{\boldsymbol{k}}}_{i\perp }+{{\boldsymbol{G}}}_{\perp }^{({m}_{0}+{\rm{\Delta }}m,{n}_{0}+{\rm{\Delta }}n)}$ that can contribute to reflection propagating outside the slab when $|{{\boldsymbol{k}}}_{\perp }^{({m}_{0}+{\rm{\Delta }}m,{n}_{0}+{\rm{\Delta }}n)}| < |{{\boldsymbol{k}}}_{i}|$. Obviously, ${{\boldsymbol{k}}}_{\perp }^{({m}_{0}+{\rm{\Delta }}m,{n}_{0}+{\rm{\Delta }}n)}$ comprises an arc of $|{{\boldsymbol{k}}}_{\perp }^{({m}_{0},{n}_{0})}|$ shifted by ${{\boldsymbol{G}}}_{\perp }^{({\rm{\Delta }}m,{\rm{\Delta }}n)}$, and this arc can be interpreted also as an arc of radius *k*_*p*_ with its centre being at ${{\boldsymbol{G}}}_{\perp }^{({\rm{\Delta }}m,{\rm{\Delta }}n)}$ (Fig. 3(c)). When ${{\boldsymbol{G}}}_{\perp }^{({m}_{0},{n}_{0})}+{{\boldsymbol{G}}}_{\perp }^{({\rm{\Delta }}m,{\rm{\Delta }}n)}=0$ as in the left of Fig. 3(b,c), the arc is simply translated. Otherwise, only a part of the arc remains because $|{{\boldsymbol{k}}}_{\perp }^{({m}_{0}+{\rm{\Delta }}m,{n}_{0}+{\rm{\Delta }}n)}|$ must be smaller than |***k***_i_| sin*θ*_*NA*_ as mentioned above (See the right of Fig. 3(b,c)). From different sets of (*m*_0_, *n*_0_) and (Δ*m*, Δ*n*), one can thus construct a set of arcs whose centres are located on a hexagonal reciprocal lattice in the case of a half-Skyrmion lattice (Fig. 1(b,e)), or on a centred-rectangular reciprocal lattice in the case of structures similar to sliced BP I (Fig. 1(c,f) and Fig. 1(d,g)).

The faint filled areas inside the sharp Kossel lines found both in the experimental and numerical Kossel diagrams are simply understood as a part of a filled circle whose radius is |***k***_i_| sin*θ*_*NA*_ and centre is at ***G***^(*m*,*n*)^: As shown in the previous discussion the in-plane component of the wavevector of the reflected light (whatever the reflectivity) is ${{\boldsymbol{k}}}_{{\rm{i}}\perp }+{{\boldsymbol{G}}}_{\perp }^{(m,n)}$, and ***k***_i⊥_ is distributed within a circle of radius |***k***_i_| sin*θ*_*NA*_. In the right panel of Fig. 3(c), the overlap of two dotted circles of radius |***k***_i_| sin*θ*_*NA*_, with the centre of the left being at ${{\boldsymbol{G}}}_{\perp }^{(m,n)}$, corresponds to the faint filled area.

**Figure 4:**
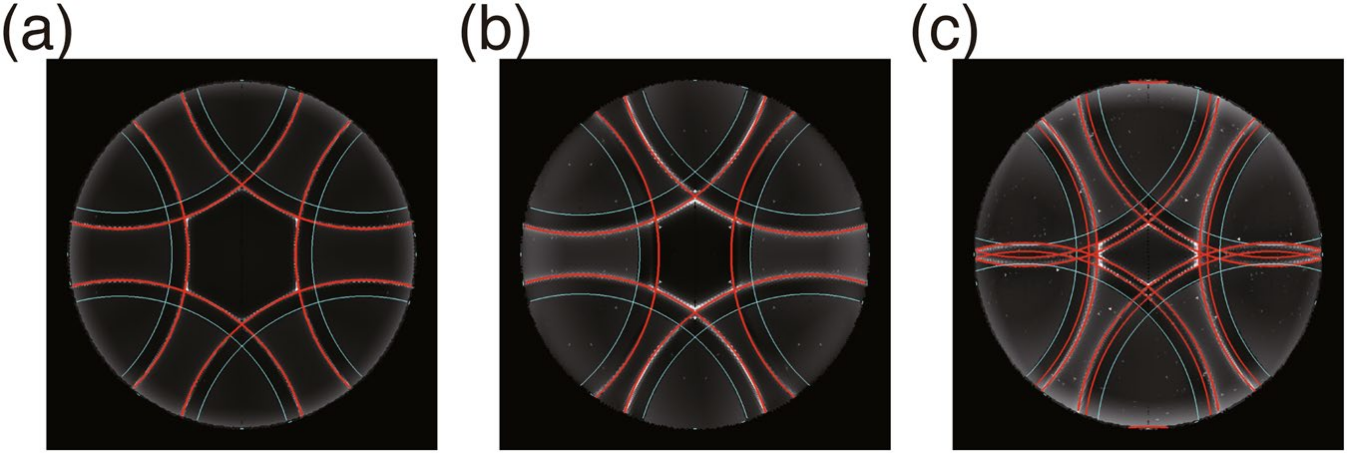
Comparison between numerically calculated and analytically obtained Kossel diagrams. (**a**–**c**) Analytically obtained Kossel lines (red) and edges of faint filled area (cyan) are superimposed onto numerically calculated ones Fig. 1(e–g), respectively.

In Fig. 4(a–c), we superimpose the Kossel lines (red) and the edges of faint filled areas (cyan) obtained analytically in the above-mentioned manner, onto the numerically calculated ones (Fig. 1(e–g)). Note that two Kossel lines with different radii, *k*_*p*_^TE^ and *k*_*p*_^TM^, are indistinguishable in Fig. 4(a,b). Double Kossel lines in Fig. 4(c) arise from the presence of two solutions for *k*_*p*_^TM^, although the intensity of one Kossel line is much smaller than that of the other in the numerical calculation (Fig. 1(g)), and double lines are hardly visible in the experimental one (Fig. 1(d)). Their locations agree perfectly with those of numerically calculated ones, which is surprising considering the fact that the above theoretical argument deals with a limiting case of weak spatial modulation of the structures.

The deviation from the above picture can be found at the intersections of two different Kossel lines; the shape of Kossel lines at an intersection is not a simple superposition of two circular arcs, and the intensities of Kossel lines emanating from the intersection are not continuous. These features are, in closer inspection, found also in experimental Kossel diagrams (See, for example, the intersection of two bright Kossel lines in Fig. 1(c)). Several previous studies^43–45^ attributed such non-trivial features of Kossel lines at the intersections to many-wave scattering, and in our cases as well, many-wave scattering is likely to be responsible for the fine structures of Kossel lines. The above theoretical argument can account for only the location of Kossel lines, not their intensities that depend of the detailed structure of the liquid crystal slab. Almost perfect six-fold symmetry in the experimental and numerical Kossel diagrams (Fig. 1(b,e)) clearly reflect the six-fold symmetry of the half-Skyrmion lattice. Less symmetry in the structures of sliced BP I (Fig. 2(c–f)) gives rise to the asymmetry in the intensities of Kossel lines (Fig. 1(c,d,f,g)). The discontinuity in the intensity of a Kossel line at the edge of a faint filled area, observed both in experimental and numerical ones (clearly seen in Fig. 1(c,d,f,g)), is attributed to the formation of partial arcs when ${{\boldsymbol{G}}}_{\perp }^{({m}_{0},{n}_{0})}+{{\boldsymbol{G}}}_{\perp }^{({\rm{\Delta }}m,{\rm{\Delta }}n)}\ne 0$ (See also the right panel of Fig. 3(c)).

## Discussion

Liquid crystals have been extensively studied as model systems that allow the investigation of structures that are predicted but inaccessible experimentally in other systems (one notable example is the realisation of Kibble-Zurek mechanism in a nematic liquid crystal)^46,47^. Our thin film of a chiral liquid crystal also serves as a model system enabling studies of topological entities including half-Skyrmions by various optical means that cannot be exploited for other systems embedding Skyrmions. We therefore believe that further optical investigation of liquid crystalline Skyrmions will shed new light on the structures and possible functions of Skyrmions in the optical regime.

As we have seen, Kossel diagrams visualise the wavevector of the modes that can be excited by incident light. Therefore Kossel lines can be regarded as the manifestation of the dispersion eigenmodes of the medium^43^, and Bragg reflections are typical examples of such eigenmodes. The same applies to angle-resolved photoemission spectroscopy (ARPES) that probes the band structures of the surface of a solid, or of an effectively two-dimensional material^48,49^. Just as ARPES played a substantial role in the discovery of three-dimensional topological insulators^50–52^, the Kossel technique could be used to investigate the dispersion properties of thin systems that exhibit non-trivial band structures such as topologically protected exotic surface states in the optical regime. As we have demonstrated, our Kossel lines are attributable solely to guided mode resonances that can be present only in systems with surfaces with outer space. Hence, dispersion modes that exist only in the presence of surfaces can be detected by the Kossel technique, and the Kossel technique could serve as additional tools for the investigation of topological photonic materials.

## Methods

### Material

The liquid crystal material is the same as that used in our previous study^16^: a mixture of a nematic liquid crystal ZhK-1289 (NIOPIC) and a chiral dopant CB15 (Merck) at 1:0.65 weight ratio. Its phase sequence is N^*^ $\mathop{\to }\limits^{26{}^{\circ }{\rm{C}}}$ BP I $\mathop{\to }\limits^{28.5{}^{\circ }{\rm{C}}}$ I (Here N^*^ and I stand for a cholesteric phase and an isotropic phase, respectively), and the lattice constant of BP I of this mixture is $\simeq 360$ nm.

### Experimental cells

How the liquid crystal cells were prepared is also the same as that in our previous study^16^. 25 × 25 mm clean cover glass plates of 150 μm thickness with planar degenerate surface alignment were used. Thin wedge cells were prepared on a hot plate at 50 °C by placing a small amount of diluted water suspension of 2 μm particles as a cell spacer along one edge of the bottom substrate. Then a tiny (0.1–0.5 l) drop of the BP material was placed onto the substrate. The drop was covered with the second plate and the plates were pressed against each other until the liquid crystal material spread and covered the whole cell area. After that, the cell was placed into a programmable hot stage for fast cooling to temperature ~1–2 K above the BP I–I transition and subsequent slow (~0.01 K/min) cooling into BP I phase.

### Microscopic observations

We used an inverted polarising microscope (Ti-U, Nikon) with ×100 oil immersion objective (*NA* = 1.4), Nikon DS-Fi1 digital camera (pixel size 3.4 × 3.4 μm). Kossel diagrams were recorded in conoscopic observation mode from a single domain with fully open illumination aperture diaphragm and fully closed eld diaphragm. Interference filters at 514.5 nm with 5 nm transmission bandwidth were used for wavelength selection. Due to low intensity of the reflected light and narrow filter bandwidth exposure times were from 4 to 30 seconds depending on wavelength and sample region.

### Numerical calculations

The orientation profiles presented in Fig. 2 were calculated by minimising the free energy functional of the liquid crystal in terms a second-rank tensor *χ*_*αβ*_ describing the orientational order. We used the same calculation code as used in our previous studies^12,53^ to find the equilibrium profiles of *χ*_*αβ*_ and their lattice constants that minimise the total free energy per unit area along the *xy* plane in which periodic boundary conditions are imposed. Further details can be found in Supplementary Methods. The calculations of the Kossel diagrams were carried out by solving the Maxwell equations for the electric field using plane-wave expansion along the in-plane direction and finite-difference discretisation along the normal direction of the cell. The reflected waves were calculated for different wavevectors of incident light, and Kossel diagrams plotted the intensities of reflected light with different wavevectors. Further technical details can be found in ref.^39^.

## Electronic supplementary material

Supplementary Information

## Data Availability

The datasets generated during and/or analysed during the current study are available from the corresponding authors on reasonable request.
