# Supplementary material for: Liquid-crystalline half-Skyrmion lattice spotted by Kossel diagrams
Source: Sci Rep. 2018 Nov 22;8:17234. doi: 10.1038/s41598-018-35514-0 (PMC6250727; doi:10.1038/s41598-018-35514-0)
Supplement: Supplementary file 1 — Supplementary Information [file 41598_2018_35514_MOESM1_ESM.pdf]

Supplementary Information for "Liquid-crystalline half-Skyrmion lattice spotted by Kossel diagrams"

by Jun-ichi Fukuda, Andriy Nych, Uliana Ognysta, Slobodan Žumer and Igor Muševič

Supplementary Methods

Calculation of the orientational order. The calculation details are already presented in Ref. 1–3, and here we show only essential points of the calculation. After appropriate rescaling of the length and the second-rank tensor order parameter for the orientational order, the rescaled free energy densities in terms of rescaled order parameter $\chi_{\alpha\beta}$ read

$$\varphi_{\rm local} = \frac{a^3}{b^4} f_{\rm local} = \tau\,{\rm Tr}\chi^2 - \sqrt{6}\,{\rm Tr}\chi^3 + ({\rm Tr}\chi^2)^2, \tag{1}$$

$$\varphi_{\rm el} = \frac{a^3}{b^4} f_{\rm el} = \kappa^2\{[(\nabla\times\chi)_{\alpha\beta} + \chi_{\alpha\beta}]^2 + \eta[(\nabla\cdot\chi)_\alpha]^2\}, \tag{2}$$

$$\varphi_{\rm s} = \frac{2q_0a^3}{b^4} f_{\rm s} = \frac{1}{2}w_1{\rm Tr}(\tilde{\chi} - \tilde{\chi}^\perp)^2 + \frac{1}{2}w_2({\rm Tr}\tilde{\chi}^2 - \chi_{\rm s}^2)^2, \tag{3}$$

and the rescaled total free energy is $F = \int dx\,dy\,\left[\varphi_{\rm s} + \int_0^L dz\,(\varphi_{\rm local} + \varphi_{\rm el})\right]$. Here L is the (rescaled) thickness, and the rescaling has been done so that the natural pitch of the helical orientational order p is 4π.

As in the previous study,[3] in the calculation of the orientation profiles (Fig. 2 and Supplementary Fig. 1(b)) and the phase diagram (Supplementary Fig. 1(a)), we chose $\kappa = 0.4$ and $w_1 = w_2 = 0.1$, which corresponds to $p \simeq 280$nm, and the anchoring strength $W_1 \simeq 0.7\times10^{-4}$J m^{-2} that can be regarded as weak. We employed the so-called one-constant approximation $\eta = 1$. This value of p, which is almost equal to the lattice constant of BP I, a, is slightly smaller than that of the liquid crystal mixture used in experiments ($\simeq$ 360nm). However, the actual dimensional value of p for given

κ depends on the choice of the material parameters, and in the calculations concerning optics, only the ratio of p (or a) to the wavelength of the incident light matters. The temperature increment $\Delta\tau = 1$ corresponds to $\Delta T \simeq 1$ K, and the profiles in Fig. 2, and Supplementary Fig. 1(b) are calculted at $\tau = -0.1$. Note that outside the temperature range we studied ($-0.5 \leq \tau \leq 0.1$) other structures are possible as shown in Supplementary Fig. 2 of Ref. 3, which are beyond the scope of the present study.

References

[1] Fukuda, J. & Žumer, S. Quasi-two-dimensional Skyrmion lattices in a chiral nematic liquid crystal. *Nature Communications* **2**, 246 (2011).

[2] Fukuda, J. & Žumer, S. Cholesteric blue phases: effect of strong confinement. *Liq. Cryst.* **37**, 875–882 (2010).

[3] Nych, A., Fukuda, J.-i., Ognysta, U., Žumer, S. & Muševič, I. Spontaneous formation and dynamics of half-skyrmions in a chiral liquid-crystal film. *Nature Physics* **13**, 1215–1220 (2017). URL `http://dx.doi.org/10.1038/nphys4245`.

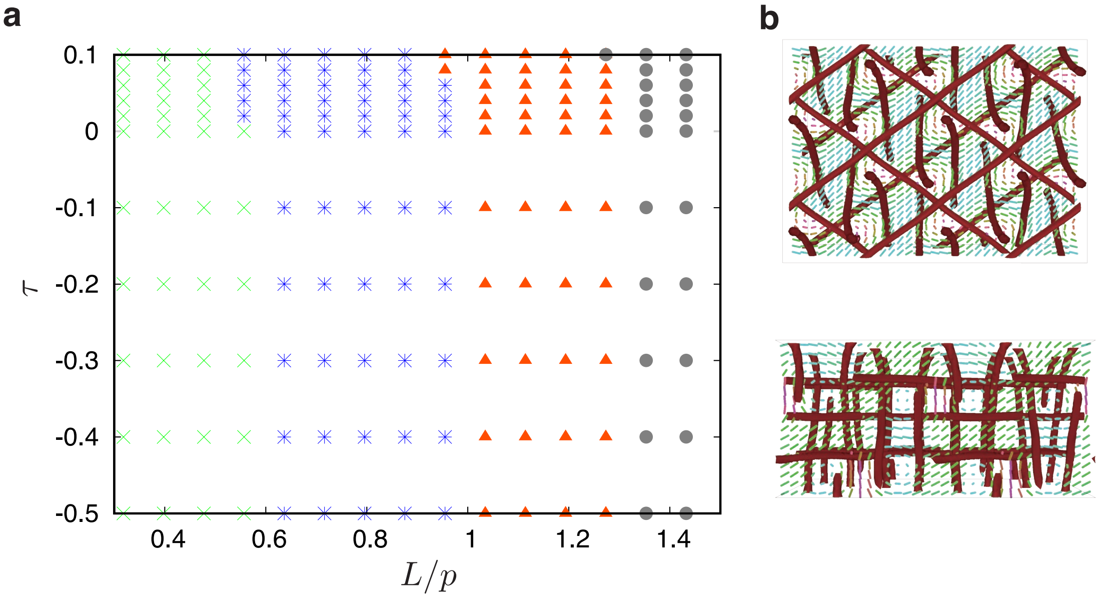

Supplementary Figure 1: **Calculated Phase Diagram.** **a**, Phase diagram with respect to the rescaled thickness L_p and temperature τ. Green cross (×) represents the structure presented in Fig. 2**a**,**b**, blue asterisk (∗) that in Fig. 2**c**,**d**, and orange filled triangle (▲) that in Fig. 2**e**,**f**. **b**, another stable thicker structure similar to a sliced BP I containing three in-plane arrays of parallel disclination lines (grey filled circle (•) in **a**).
